# Shaping EU Plastic Policies: The Role of Public Health vs. Environmental Arguments

**DOI:** 10.3390/ijerph16203928

**Published:** 2019-10-16

**Authors:** Linda Mederake, Doris Knoblauch

**Affiliations:** Ecologic Institute, 10717 Berlin, Germany

**Keywords:** plastics, plastic policies, plastic pollution, EU, case study research, qualitative content analysis, policy formulation, EU Plastics Strategy, single-use plastics

## Abstract

Few other environmental problems have received as much public attention and criticism in recent years as plastic pollution. Accordingly, in recent years, a number of plastic policies have been adopted at the national and supranational level in the EU and worldwide. In the U.S., health risks were repeatedly raised in the decision-making process of these policies and scholars have pointed out the crucial role of these arguments for the adoption of plastic policies. Hence, this article uses a structuring qualitative content analysis to investigate the parliamentary debates of two recently adopted plastic policies in the EU—namely the EU Plastics Strategy and the Single-Use Plastics Directive—and to assess the relevance of public health and environmental arguments for the EU debate. The analysis reveals broad support for plastics regulation among Members of the European Parliament, who most often use environmental arguments to corroborate their support for the policies in question. In contrast, health concerns do not seem to be crucial for the adoption of plastic policies in the EU.

## 1. Introduction 

Plastics are subject to fierce criticism by the public. In fact, hardly any other environmental problem has received as much attention and criticism in recent years as plastic pollution and surveys show, especially for Western Europe, that marine plastic pollution is perceived as one of the most serious environmental problems worldwide [1,2].

While scholars have found much evidence on the harmful effects of (macro)plastics on marine species due to entanglement, smothering and ingestion [3,4,5] and provided some evidence on the negative ecological impacts of micro- and nanoplastics on flora and fauna [6,7,8,9,10], research regarding the potential effects of micro- and nanoplastics on human health is still in a very early phase [11,12,13,14]. Yet, despite many open questions that researchers continue to pursue, it is not too early for political responses against plastic pollution [15,16], in particular because human health risks and environmental harm are not the only factors in favor of adopting plastic policies (see further below). Accordingly, in recent years, a number of plastic policies have been adopted at the national and supranational level in the EU and worldwide. 

These policies include regulatory approaches controlling production and sales of plastic bags, single-use plastic items and microbeads [17,18,19,20,21], circular economy and waste policies (including recycling and recyclates) [22,23], import bans for plastic waste [24], as well as legislation focusing on sea-based sources of (marine) plastic pollution [21,25]. 

At the international level, in May 2019, the Parties to the Basel Convention on the Control of Transboundary Movements of Hazardous Wastes and their Disposal adopted stricter rules on the trade of plastic waste [26]. A new classification is intended to ensure that hazardous and polluted plastic waste may only be shipped with the consent of the exporting and importing country. This makes it more difficult to dispose of plastic waste in countries with lower environmental standards (and lower labor costs).

For the U.S. Microbead-Free Waters Act of 2015, Strifling (2016) [27] suggests that one of the explanatory factors for the uncontested adoption of the Act has been the emphasis on public health aspects of the proposed legislation. This is to say that the Act was explicitly positioned as a public health bill, rather than an environmental protection bill, as per its classification on the Congressional website, where every bill and resolution is assigned a single policy area term which best describes the entire measure. “Health” and “environmental protection” are among the thirty-two available policy terms [27]. This seems in agreement with the proposition put forward by Aden (2012), who, in his introductory work on environmental policy, highlights: “Environmental policy objectives have a particularly high chance of being implemented, if they also aim to protect human health” [28] (p. 54, translation by the authors).

Against this background, public health concerns could also be considered as a central explanatory factor for the recent adoption of plastics regulation at the EU level. However, there has not been any social science research investigating this assumption yet. To fill this gap, this article examines the following research question: Which types of arguments are used by parliamentarians in the European debate on plastics regulation?

To address this question, this article analyses four plenary debates of the European Parliament on the two most recently adopted plastic policies in the EU, namely the European Strategy for Plastics in a Circular Economy [22], hereafter the EU Plastics Strategy, and the Directive (EU) 2019/904 on the reduction of the impact of certain plastic products on the environment [21], hereafter the Single-Use Plastics Directive (or SUP Directive). This article uses a qualitative content analysis to assess the relevance of public health arguments for the adoption of plastic policies at the EU level. This allows the following hypotheses to be tested:

**Hypothesis** **1.** Health concerns and environmental concerns are the central arguments in support of plastics regulation and are of a similar importance in the European debate on plastics regulation.

**Hypothesis** **2.** Members of the parliamentary group “The Greens/European Free Alliance” put forward environmental concerns as arguments more frequently than other parties do.

To formulate our first hypothesis, we follow Aden (2012), who outlined that, since its historical starting point in the 19th century, the main reasons to implement environmental policies have been environmental concerns (i.e., environmental destruction) and human health risks [28] (p. 16). 

There is also a moral or ethical dimension to the preservation of ecosystems. With regards to plastics, this argument would, for instance, entail the following reasoning: “we should prevent litter from entering the ocean because it does not belong there, irrespective of whether there is an economic argument for doing so or that major impacts from plastics or microplastics cannot be proven” [29] (p. 108). In other words, an unpolluted ocean is considered to have value in and of itself. Motivations for nature conservation linked to ethical concerns and altruistic preferences can be categorized under three so-called “non-use values” [30]:

1. The existence value (satisfaction derived from the simple knowledge that an ecosystem exists and will continue to exist),
2. The bequest value (satisfaction derived from ensuring that intact ecosystems are passed on to future generations) and
3. The altruistic value (satisfaction derived from ensuring that an ecosystem is available to other people in the current generation).

A frequent line of conflict is the one between environmental protection and economic interests: Environmental policy measures are often met with resistance from companies, industrial sectors or trade associations that fear high costs if they have to adapt their products or plants to new environmental policies. In the EU, competitive disadvantages compared to suppliers from countries or regions outside of the Union often serve as an argument against national or European environmental protection measures. On the other hand, environmental policy can also generate impulses for economic growth [28] (p. 48f). In fact, waste policy is characterized by complex economic interest structures [28] (p. 41): plastic waste can still have an economic value and in this case, can be treated and sold as a resource. Policies that establish viable end markets for plastic waste, that stimulate desirable products and processes and remove barriers to investment, could thus enhance political stability for resource recovery industries. This also strengthens the independence from global market impacts [31]. At the same time, waste management has become an attractive market in a number of states, as waste producers have to pay for waste disposal according to state regulations. Accordingly, different forms of reuse, recycling and disposal compete with one another.

While environmental policy was established as a policy field in the late 1960s and early 1970s, already in the 1980s, the idea of replacing regulatory requirements with market-oriented policy instruments gained momentum. Market-oriented incentives are steering instruments designed to persuade actors to change their behavior in line with environmental policy objectives by offering the prospect of economic benefits rather than by imposing regulatory sanctions [28] (p. 81f), a line of reasoning that is still frequently used today.

As one explanatory factor for a potential stronger focus on environmental concerns in the European debate on plastics regulation than in the U.S. debate, we test the second hypothesis. As Green parties are more popular in the EU and are also represented in the European Parliament (50 out of 751 seats after the European elections in 2014 [32]), they are more able to move the European debate towards environmental issues than is the case in the U.S. context.

The article is structured as follows: Section 2 explains the methodology used for the qualitative content analysis of EU plenary debates on recently adopted plastic policies and introduces the coding guideline. Section 3 presents the results, indicating a broad approval of the proposed policies with an emphasis of environmental arguments. Health arguments play a lesser role in the statements of the parliamentarians. The Discussion critically reflects on the findings, links them back to existing research and indicates needs for future research. The Conclusion summarizes the findings and points out the implications of the results for the adoption of future policies.

## 2. Materials and Methods

We use qualitative content analysis based on Mayring [33] to study the types of arguments used by Members of the European Parliament (MEPs) in plenary debates on two recently adopted EU plastic policies. Qualitative content analysis is a mixed methods approach to analyzing documents following a systematic procedure. The assignment of categories to text passages is a qualitative step, while working through many text passages and analyzing the frequencies of categories is a quantitative one [33,34]. As such, this method is well suited for a theory-guided text analysis, whereas it is less suitable if a research question requires an explorative-interpretive or holistic approach in which case working with categories and a step-by-step approach could turn out to be a restriction. The method also faces some limits with regards to replicability (see below on inter-coder reliability) and generalizability. Yet, the strength of the approach is the ability to structure a vast amount of text, while text passages are still always interpreted within their context. This characteristic of the method is key for the present analysis. For instance, a qualitative content analysis allows the identification of irony in parliamentarians’ statements or arguments that would have slipped through a quantitative, lexical search based on word counts [33]. 

The empirical basis (“corpus”) for the study are minutes of plenary debates of the European Parliament (EP) on the EU Plastics Strategy and the SUP Directive. The EU Plastics Strategy is a Commission Communication presented in January 2018 on which the European Parliament adopted a resolution in February 2019. The EU Plastics Strategy outlines environmental and economic challenges posed by plastics throughout the value chain. It contains commitments for action at the EU level by the Commission and a list of measures recommended to national authorities and industry. With this strategy, the EU Commission aims to protect both EU residents and the environment. At the same time, the Commission wants to boost the plastics recycling industry in Europe and create a market for recycled plastics [22]. The SUP directive contributes to the objectives set out in the EU Plastics Strategy. The directive aims to reduce plastic marine litter from single-use plastic items and fishing gear by, among other smaller measures, banning certain single-use plastics (such as plastic cutlery and straws) and reinforcing the polluter pays principle, in particular for tobacco, through the introduction of extended producer responsibility (EPR). The directive entered into force on 2 July 2019 and needs to be transposed into national law in the EU member states by 3 July 2021 [21].

The EP plenary minutes are analyzed by means of a structuring qualitative content analysis with the help of nominal contents-based categories. The categories were developed deductively and then pilot tested with about 15 percent of the material. The category system was found to be comprehensive. Thus, no additional category was added after the pilot test. We use MAXQDA 10 (by VERBI Software GmbH in Berlin, Germany) for data coding. The content analytical unit consists of (at least) a partial sentence and up to a paragraph of the parliamentarians’ speeches (or four sentences, if paragraphs are very short, i.e., one or two sentences each). 

A particular challenge for the analysis is the multilingualism of EU debates. Hence, our article is one of the first that covers such a brought set of languages in a qualitative content analysis. To enhance inter-coder reliability, the first and second author independently coded the majority of the material (statements in German, English, French and Spanish) and results were compared. The rest of the material was coded by native speakers and cross-checked by the authors or the authors coded a translation of the statements and native speakers (or speakers with high proficiency in the respective language) cross-checked the coding with the original statement. Seventeen statements out of 198 statements, i.e., those in Croatian, Finnish, Gaelic, Maltese and Slovakian, were not coded due to missing language competencies.

Apart from the types of arguments, we also coded the speakers’ affiliation to a certain parliamentary group or committee or membership in the European Commission/Council. This allows for an additional analysis based on cross-tables. The parliamentary groups include ALDE (The Alliance of Liberals and Democrats for Europe), ECR (European Conservatives and Reformists), EFDD (Europe of Freedom and Direct Democracy), ENF (Europe of Nations and Freedom), EPP (European People’s Party), The Greens/EFA (The Greens/European Free Alliance) and S&D (Progressive Alliance of Socialists and Democrats). Additionally, the statements by independents (NI) and representatives from three committees (agriculture/AGRI, environment/ENVI, fisheries/PECH) were coded. The results of NI, the committees and the Council are not analyzed in detail due to the small number of coding units. 

The arguments in favor of and against plastics regulation that were formulated based on the literature on rationale for the adoption of environmental policies, as outlined above, were divided into the following categories: 

Category 1: “pro regulation” comprises five argument categories, namely 1 A no specific reasons, 1 B environmental concerns, 1 C human health concerns, 1 D economic considerations and 1 E non-use values. 

Category 2: “pro regulation, if certain conditions are met” includes three argument categories, namely 2 A conditions are not specified, 2 B focus on economic incentives, 2 C additional measures necessary. 

Category 3: “contra regulation” comprises another five argument categories, namely 2 A no specific reasons, 2 B lack of evidence for environmental harm, 2 C lack of evidence of harm to human beings, 2 D economic considerations and 2 E problem is not addressed in the right way.

The coding guideline can be found in Appendix A.

## 3. Results

In total, 463 text units were coded, 244 units for the two debates on the Plastics Strategy and 219 units for the two debates on the SUP Directive. All four debates were characterized by a very broad consensus of parliamentarians that regulations are needed and that the proposed policies provide a step in the right direction. The category frequencies speak for themselves: Around 70 percent of the coding units fall under Category 1 (pro regulation) and another 22.2 percent fall under Category 2 (pro regulation with reservations). Less than 8 percent of the units are coded as Category 3 (contra regulation).

### 3.1. Arguments in Favour of and Against Regulation

**Figure 1:**
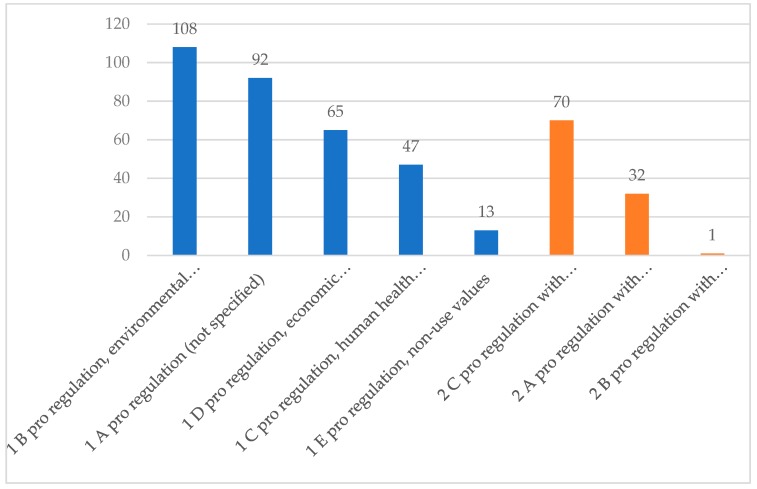
Coding units per sub-category for category 1 (pro regulation) and category 2 (pro regulation with reservations).

With regard to Category 1 (pro regulation), all four arguments included in the coding system were mentioned by parliamentarians (see Figure 1). Among them, Category 1 B (pro regulation due to environmental concerns) was most frequently mentioned (108), followed by Category 1 A (pro regulation without providing specific reasons, 92). 

For Category 2, 2 C (pro regulation, but additional measures necessary) was coded most often, with 70 coding units, followed by 2 A (pro regulation with reservations) with 32 coding units, while 2 B (pro regulation, but regulation should focus on economic incentives) was coded only once. 

**Figure 2:**
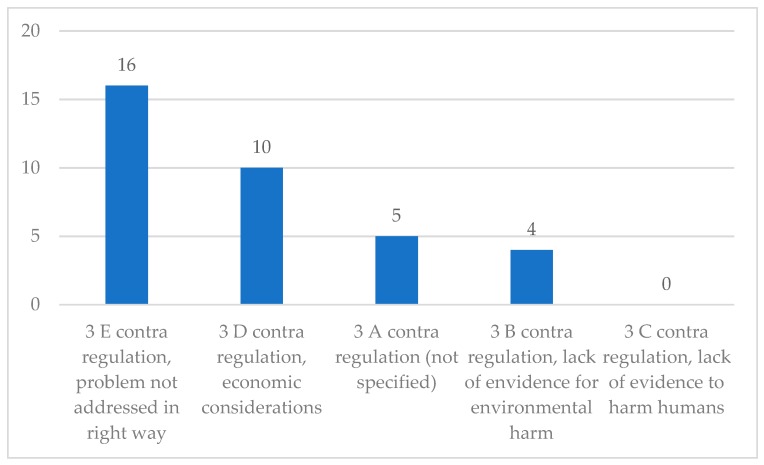
Coding units per sub-category for Category 3 (contra regulation).

The argument most frequently put forward against regulation is that the proposed regulations do not address the problem in the right way (3 E; 16), as illustrated in Figure 2. Nevertheless, the parliamentarians arguing this usually acknowledged the plastic problem and are not arguing against any kind of action. None of the parliamentarians argued against regulation due to a lack of evidence to cause harm to human beings, but a lack of evidence to cause environmental harm is mentioned four times. 

Arguments against plastics regulation were mentioned much more often in the two debates on the SUP Directive (26) than in the debates regarding the Plastics Strategy (9) (see Table 1). While the differentiation between the two policies was not at the core of this research, it is nevertheless noticeable that health arguments were proportionally mentioned more often in the debates on the Plastics Strategy, while the pro categories providing no specific arguments (or new arguments), namely 1 A and 2 A were coded more often for the SUP Directive.

The analysis of overlapping coding units reveals that health concerns are often mentioned together with environmental concerns, namely in 34 out of 47 coding units for 1 C. Economic arguments coincide with environmental arguments much less often (overlaps in 23 out of 65 1 D coding units).

### 3.2. Differences in Party Group Affiliation Regarding Argumentation

Arguments pro regulation outnumbered arguments against regulation by far for all party groups except two (see Table 2). For EFDD, a Eurosceptic and populist political group, 38.5 percent of the coding units include statements against regulation. For ENF, a nationalist far-right group, as much as 50 percent of the coding units include statements against regulation. For two party groups (The Greens/European Free Alliance and ECR) all statements are in favor of regulation, yet with a certain amount of reservations. Proportionally, members of the European United Left/Nordic Green Left (GUE/NGL) have voiced reservations most often, namely in 50 percent of their coded statements. 

Unsurprisingly, the highest amount of unconditional support for the policies comes from the European Commission. A total of 97.5 percent of the coding units fall in category 1 (pro regulation).

**Figure 3:**
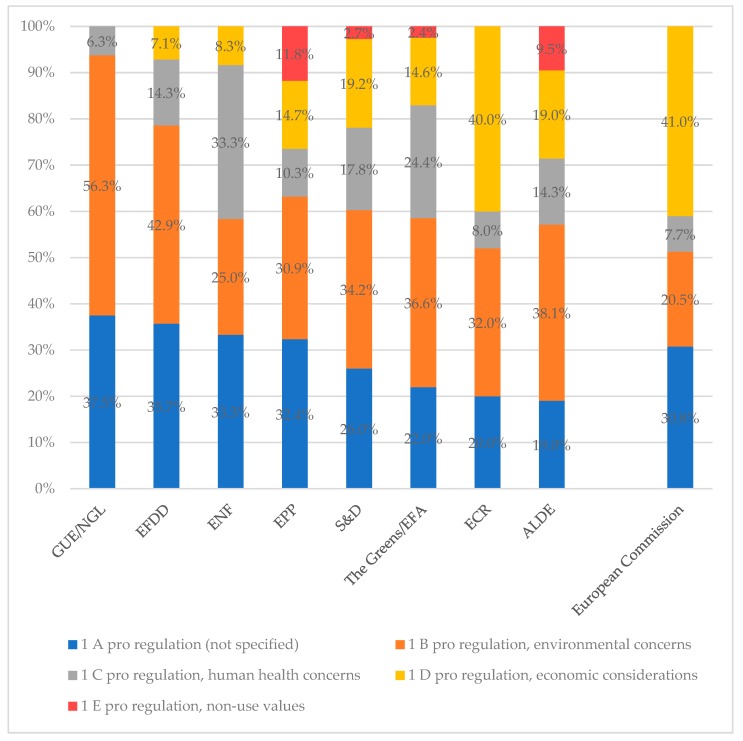
Sub-category frequencies for category 1 (pro regulation) per parliamentary group and for the European Commission.

With regard to the arguments in favor of regulation, environmental concerns were the most prominent argument for members of ALDE, EFDD, The Greens/EFA, GUE/NGL and S&D, while for ENF and EPP, statements in favor of regulation without providing a specific reason were more prevalent (see Figure 3). Members of ECR and the Commission emphasized economic considerations most often. At the same time, the percentage of arguments stating environmental concerns is particularly low for the Commission (lower than for any of the party groups). 

The argumentation line of GUE/NGL members seems most clear-cut, with the highest percentage of coding units in Category 1 for environmental concerns (56.3 percent) and the lowest for health concerns (6.25 percent) and zero coding units for economic considerations. 

ENF members (33.3 percent) as well as members of The Greens/EFA (24.4 percent) raised health concerns most often. Non-use values (Category 1 E) are most prominent among members of EPP (11.8 percent) and ALDE (9.52 percent).

## 4. Discussion

In our Hypothesis 1, we assumed that health concerns and environmental concerns would be central arguments in support of plastics regulation and were of similar importance in the European debate on plastics regulation. However, our results do not confirm this hypothesis. Environmental reasons were named 108 times, more than twice as often as health concerns, which were raised only 47 times. Even economic reasons for introducing the legislation were named more often than health reasons, 65 times in total. In order to provide insights into why this is the case, we first take a closer look at the specific content of the arguments and then discuss potential explanatory factors for the differences between the U.S. and the EU.

The environmental concerns related to plastics that can be identified in the literature include the problems of habitat damage [29] (p. 94), entanglement through plastic particles or their ingestion [3,4,5,29,35], as well as the fact that fossil fuel-based plastics further contribute to CO_2_ emissions and climate change [36]. Our analysis shows that all of these environmental concerns are shared and voiced by decision makers and politicians who argued for plastics regulations. For instance, Tilly Mertz (The Greens/EFA) argued for the Plastics Strategy: “Between 150,000 and 500,000 tons of plastics end up annually in the sea, with devastating effects on the ecosystems of the oceans. Animals such as fish, tortoises and birds catch or suffocate in plastics” (translation by the authors). During the debate about the SUP Directive, Seán Kelly (EPP) stated that “[…] an awful lot of plastics—as has been said—finishes up in the seas, finishes up in the bellies of fish, killing them and polluting”. Doru-Claudian Frunzulică (S&D) expressed in writing: “The way plastics are produced, used and disposed of today has devastating environmental, climate and economic drawbacks and potential negative health impacts on both humans and animals” ahead of the debate about the Plastics Strategy. In addition, Mark Demesmaeker (ECR) pointed out: “This is not only good for the environment, but also for the economy and for the climate, because we prevent CO_2_ emissions. With every ton of plastics we recycle, we take a car off the road” (translation by the authors).

Human health concerns that can be found in the literature include concerns about chemical exposure [29] (p. 102/3), health concerns associated with poorly managed waste collection and treatment [29] (p. 101) and last but not least, arguments related to the threats posed by microplastics and nanoplastics to food safety [29] (p. 102). Our analysis shows that MEPs voiced these human health concerns in the plenary debates with the exception of the health concerns associated with poorly managed waste collection and treatment. Sirpa Pietikäinen (EPP), for instance, argued for the SUP Directive: “What I mean is that plastics—and the microplastics and nanoplastics which are actually the most severe problem—do come from other sources also. They are used in detergents, cosmetics, packaging and fertilisers, and so we eat them because they get into a very tiny format”. Moreover, Christelle Lechevalier (ENF) highlighted: “Similarly, some so-called “biodegradable” plastics do not disappear. Plastic breaks down into microparticles or endocrine disrupters that are found absolutely everywhere. Water, food, but also atmosphere, toys, floors, furniture, make-up, everything goes through it. In short, dramatic consequences on health and especially during the first stages of life, when our hormones play as important a role as genes in physiological development. Here, the impacts can be irreversible for our children”. 

Regarding the economic concerns, loss of revenue for some industries like fisheries or the seafood industry etc. [29] (p. 95) are arguments for plastics regulation as are the negative effects of augmenting pollution on the tourism sector [29] (p. 106). At the same time, plastics regulation promoting more recycling can also create economic opportunities. For instance, it is argued that the EU might become independent from the world market [31] or that generally business opportunities arise for recyclers [31]. Again, these arguments are used not only by scientists and other experts, but also by the decision-makers bringing forward their arguments in the plenary debate. Seán Kelly (EPP) elaborated during the debate on the Plastics Strategy that “We still use single-use plastic cups for our coffees, plastic straws in our drinks, single-use plastic water bottles and plastic cutlery in many of our cafes and takeaways. This leads to pollution across the continent, particularly in our coastal areas and marine environments, where it has a massive negative impact on our maritime and fisheries sectors, which has obvious implications for human health and indeed tourism”. Doru-Claudian Frunzulică (S&D) put forward in writing: “I very much welcome this report, which tackles successively the need to move from design for recycling to design for circularity, the creation of a genuine single market for recycled plastics, prevention of plastic waste generation, as well as innovation and global action”. And Françoise Grossetête (EPP) added: “China’s decision to ban imports of European plastic waste offers us an opportunity to create the conditions for a genuine internal market for recycled materials”.

This closer look on the content of specific arguments reveals that politicians presented almost all the arguments that are outlined in the scientific literature, which is not always the case in other debates. Thus, the debate was obviously very broad. There is only one exception: regarding the so-called non-use values [30], three different categories can be distinguished, namely the (1) the existence value, (2) the bequest value and (3) the altruistic value. Of these three, only the bequest value was mentioned explicitly: Tunne Kelam (EPP), for instance, warned: “We need an ambitious goal to save the next generation from drowning in a new ocean of waste”.

Apart from the arguments discussed in the literature, we also found further arguments that were put forward in the plenary debates: several times, it was mentioned that it was the society’s will to regulate plastics and that the EU did act because of the citizens and the public pressure. For instance, Frans Timmermans, First Vice-President of the Commission, underlined that “There is a huge sense of urgency in European society that this needs to be done and it needs to be done now”. Karmenu Vella, Commissioner for Environment, Maritime Affairs and Fisheries highlighted: “this legislation is one of the most called for and also one of the most supported EU initiatives among European citizens. Our main task now is to ensure that the ambitious measures enshrined in it are also quickly implemented in practice”. Frédérique Ries, the rapporteur for the SUP Directive, said: “And for my team, I would add, the citizens are essential, these millions of mobilized Europeans who pressed us, encouraged us to place the cursor very high for the oceans, of course, for the environment, but above all for us and for our children” (translation by the authors). Finally, James Nicholson (ECR), during the Plastics Strategy debate stated: “I think that it is a very opportune moment to be debating this today, at a time when citizens not only in my own constituency, but across Europe and indeed the world are more conscious of the impact which plastic use is having on our environment. There is a clear momentum in tackling these issues, and now is the time to act”.

Another argument put forward by the EU Commission of why the EU is going for plastics regulation is relation to the Sustainable Development Goals (SDGs). Frans Timmermans mentioned that the legislative proposal was also “a concrete demonstration of the EU’s broader commitment to the UN Sustainable Development Goals”. Karmenu Vella added that the EU should become “the world leader when it comes to the most sustainable plastic policy”.

Finally, another argument that was brought forward was that member states alone could not handle the plastic problem but that the problem needs to be addressed at EU level. At the same time, other parliamentarians argued that the EU alone could not solve the problem, but that there is a need for a binding international target to reduce plastic pollution, i.e., that the problem should be addressed at the international level. Soledad Cabezón Ruiz (S&D) also mentioned that the municipalities needed to play an active part with regards to plastics regulation.

More rarely used arguments were that the regulation is supported because it puts obligations on producers. Peter van Dalen (ECR) mentioned that he wanted to reduce the dependence on Saudi Arabia: “We are reducing our dependence on oil-producing countries such as Saudi Arabia, which is a Salafistic country” (translation by the authors) and Karmenu Vella, Member of the Commission stated that the regulation was needed to avoid the fragmentation of the single market. 

In addition to the arguments brought forward to adopt the regulations, we also investigated the reservations raised by parliamentarians. It is noteworthy that this closer look at the context of statements coded as 2 A and 2 B reveals that very few genuine reservations were stated during the debates, while many MEPs called for more ambition in general (most 2 A coding units) or named specific additional EU measures (2 C). Only one statement was coded as 2 B “pro regulation, but regulation should focus on economic incentives”, although one would expect this argumentation to come up more often, e.g., in statements of liberal MEPs. 

The additional measures MEPs wanted to see included in the legislation are measures to

- Set concrete and binding targets;
- Introduce the polluter-pays principle;
- Reduce food packaging;
- Discourage multi-layer packaging;
- Encourage easier-to-recycle plastics;
- Ban plastic bags;
- Reduce microplastics in products (e.g., in cosmetics and detergents);
- Reduce microplastics from tires and textiles;
- Address a change in lifestyle, in behavior or a cultural change e.g., with regard to plastic use and waste disposal;
- Support a circular economy;
- Undertake a regular monitoring of the proposed legislation;
- Support innovation and technological development;
- Promote the replacement of single-use plastic products instead of prohibiting them;
- Implement strict bans on single-use plastics if there is an alternative at hand;
- Avoid any replacement of plastic products with supposedly biodegradable products;
- Raise awareness in the industry;
- Strongly regulate plastic manufacturers;
- Support the exchange between manufacturers and recyclers as well as between manufacturers and consumers;
- Support exchange between designers and recyclers;
- Environmentally educate consumers;
- Take a more consistent approach between chemical and waste rules;
- Reduce plastics in cigarettes;
- Reduce hazardous substances in plastics;
- Set minimum standards for recycled content in the Ecodesign Directive;
- Create an internal market for recycled materials;
- Reduce the plastic use within the European Parliament;

Although the abovementioned list of measures is not exhaustive, it is already quite impressive, suggesting that more legislation is to be expected, also because of the high pressure the EU is facing from citizens, as expressed several times during the debates. Reducing (food) packaging was mentioned regularly. Of course, out of the additional measures that were mentioned by the parliamentarians, some contradicted each other—some want stricter regulation, others less, some want the industry to be targeted, others the consumers.

Regarding the type of instruments that should be used, a number of parliamentarians also stated their opinion explicitly. Some favored a regulatory over a market approach, arguing against economic instruments, some said that taxes or levies are useful incentives for the economy. One MEP called for a framework legislation, leaving the concrete implementation to EU member states. Finally, one Parliamentarian stated that legislation should only be adopted if social equity is ensured throughout its implementation.

The arguments against regulation stemmed almost exclusively from Italian and UK-representatives, and predominantly from representatives of the ENF and the EFDD. MEPs from these two parliamentary groups see national economic interests threatened because they have industries working particularly in the field of oxo-degradable plastics, which will be banned by the SUP Directive. Along these lines, another argument against the regulation was that it would not only lead to job losses, but furthermore, supposedly break EU law (by not following the appropriate procedures under REACH when banning oxo-degradable plastics). In particular, John Stuart Agnew (ENF) stated: “I am concerned that the EU is fraudulently harassing British innovation in degradable plastics. Parliament has not yet been made aware that, if we accept the reference to oxo-biodegradable plastics in Article 5, we would be evading the European Union’s own rules for banning substances”. Moreover, Bill Etheridge (EFDD) put forward: “We all agree that we need to make changes and improve the environment, and this is a particularly important issue. However, manufacturing and production always need time to change. You stressed several times in your remarks: Urgency, urgency, urgency. Would you not accept that the speed of the motion that we have before us will actually lead to job losses? And, if it does lead to job losses, how many are acceptable? 500? 1000? 10,000? Where does it stop? Where do we take into account the needs of manufacturers, as opposed to the prerogative for change?”.

The findings with regard to our second hypothesis that members of the parliamentary group The Greens/European Free Alliance put forward environmental concerns as arguments more frequently than other parties do provide a first indication of why environmental arguments are more prominent in the European debate than in the U.S.: Of the 22 coded statements by Green MEPs, 15 coding units were coded as 1 B, compared to 93 coding units in 159 coded statements for MEPs from other parliamentary groups. The results confirm that Green MEPs use environmental arguments more frequently than MEPs from other parties do (68.2% vs. 58.5%). The Greens, who are more prominent in the EU compared to the U.S. context, are thus more likely to move the European debate towards environmental issues than is the case in the U.S. In contrast to environmental issues, health as an issue is not connected to a particular party.

Another possible explanation for why health arguments have not been raised as often by MEPs is the fact that to date, there is no proof that (micro-)plastics actually affect human health [37]. This is despite the fact that several recent studies pointed to the potential risks for human health (e.g., [12,38,39,40]), or stated that the effects of microplastics on human health are still unknown (e.g., [35,41,42]). Against this background, it is even more striking that health arguments were predominant in the U.S. debate. This might be due to the fact that the U.S. legislation in question was on microplastics, while in the EU, it had a much broader scope. Alternatively, it might be the current political climate in the U.S., where environmental issues are not very prominent on the national agenda, and thus, health arguments were used as means to avoid an environmental debate. However, investigating this new hypothesis would require further in-depth research and a systematic comparison between the U.S. and EU debates.

What we can see from our analysis of the EU debate is that health concerns are often mentioned together with environmental concerns, namely in 34 out of 47 coding units. This is in congruence with Aden’s (2012) argumentation that “Environmental policy objectives have a particularly high chance of being implemented, if they also aim to protect human health” [28] (p. 54, translation by the authors). Yet, in the EU, public health arguments are not necessary to get plastic policies adopted. It rather seems that environmental arguments form the core of the arguments presented by most MEPs, while health arguments are only mentioned in addition, to reinforce the claim for regulation.

To conclude, plastics are currently widely discussed and are so prominent on the political and societal agenda that the issue could become a policy field of its own. Plastics are a subject that relates to the environment and to health aspects but also resource use, recycling, consumerism, biodiversity, climate mitigation, etc. In our analysis, we observe that—unlike in the U.S.—the topic of plastics is clearly framed as an environmental issue in the EU debate. For instance, Jo Leinen (S&D) explicitly stated during the debate: “Congratulations to this European Directive against single-use plastics. As you have seen, there is a great deal of public support for what we are doing here. And it is also something visible in the European Union’s environmental policy” (translation and highlighting by the authors). And Peter Liese (EPP) summarized: “For today, though, this is a very, very important first step: we are strengthening the circular economy, we are doing something to prevent the littering of the seas, and I have rarely seen that environmental legislation is viewed so unreservedly positively by the public, and that is why we should adopt it with a very large majority” (translation and highlighting by the authors).

Our findings lead us to conclude that plastics regulation is perceived as part of environmental policy in the EU. However, further research is needed in order to systematically analyze whether this is also the case in other EU debates that relate to plastics. An in-depth comparison between the U.S. and the EU policy debates could offer an opportunity to explore the explanatory power of factors that we identified for the differences in the EU and the U.S. Moreover, it could shed light on further reasons behind the fact that plastics seem to be perceived as a health issue in the U.S., while as an environmental topic in the EU. Additional studies could also examine whether citizens, scientists or business actors in the EU support plastic regulation for the same reasons that were most prominent among MEPs.

Another important issue for future research would be to analyze the particular role of the European Commission as the qualitative content analysis revealed the interesting fact that the Commission emphasized economic considerations more often than parliamentarians, while the percentage of arguments stating that environmental concerns was particularly low for the Commission (lower than for any of the party groups). While an in depth analysis of these findings was beyond the scope of this article, these results should be further investigated in an additional study.

## 5. Conclusions

This study set out to analyze debates in the European Parliament on two recently adopted plastic policies, namely the Plastics Strategy and the SUP Directive, by means of a qualitative content analysis. For the analysis, statements were coded for types of arguments in favor and against plastics regulation. The European multilingualism constituted a challenge for this analysis, which is why few scholars have used discourse analysis or qualitative content analysis to investigate European debates to date. 

The results of this study show very broad support for plastics regulation in the European parliament in general and for the two policies in particular. Yet, the first hypothesis that environmental and health arguments constitute central arguments in support of plastics regulation and that both are of similar importance in the European debate on plastics regulation was not confirmed. Instead, environmental arguments were more frequently used to underpin the call for plastics regulation. This highlights that MEPs perceive plastic pollution first and foremost as a serious threat to environment and ecosystems’ health. In addition, parliamentarians have voiced economic arguments more often than health arguments. 

Given the broad support for plastics regulation, differences between party groups are relatively small. Nevertheless, the findings confirm the second hypothesis that Green MEPs voice environmental concerns more frequently than other parliamentarians do. Arguments against regulation were put forward mostly by ENF and EFDD members that fear economic consequences for industries in their constituencies/countries. The Commission strongly emphasizes economic opportunities, in fact, more so than parliamentarians do.

The discussion further showed that parliamentarians used a number of additional arguments (in addition to the arguments deduced from literature) to argue in favor of regulation. These include the strong public pressure, the EU’s claim for leadership in the environmental policy field, the need to avoid fragmentation of the single market as well as the wish to decrease the dependence on other states for raw material supply.

For the adoption of future policies, the findings highlight that environmental concerns are a major push factor for the adoption of plastic policies; in contrast to the U.S., it does not necessarily need health arguments to facilitate adoption. Furthermore, the wide range of arguments used allowed each and every parliamentarian to make a statement in favor of the regulation that is consistent with their general political mindset and values. Thus, it becomes very difficult for vehement opponents to argue against it.

Overall, the wide range of arguments used in favor of plastics regulation, the strong public pressure, and the numerous additional measures that MEPs proposed make it very likely that further EU plastic legislation will follow in the near future. 

## Figures and Tables

**Table 1:** Category frequencies per regulation.

	1 A	1 B	1 C	1 D	1 E	2 A	2 B	2 C	3 A	3 B	3 C	3 D	3 E	∑
SUP Directive	49	50	14	21	10	18	1	30	5	3	0	8	10	219
Plastics Strategy	43	58	33	44	3	14	0	40	0	1	0	2	6	244
**∑**	92	108	47	65	13	32	1	70	5	4	0	10	16	463

**Table 2:** Category frequencies per parliamentary group of the European parliament and for the European Commission.

Parliamentary Group	1 Pro Regulation	2 Pro Regulation with Reservations	3 Contra Regulation
ALDE	70.0%	26.7%	3.3%
ECR	86.2%	13.8%	0.0%
EFDD	53.8%	7.7%	38.5%
ENF	42.9%	7.1%	50.0%
EPP	61.3%	35.1%	3.6%
The Greens/EFA	82.0%	18.0%	0.0%
GUE/NGL	47.1%	50.0%	2.9%
S&D	78.5%	17.2%	4.3%
European Commission	97.5%	2.5%	0.0%

The full names of the parliamentary groups are The Alliance of Liberals and Democrats for Europe (ALDE), European Conservatives and Reformists (ECR), Europe of Freedom and Direct Democracy (EFDD), Europe of Nations and Freedom (ENF), European People’s Party (EPP), The Greens/European Free Alliance (The Greens/EFA) and Progressive Alliance of Socialists and Democrats (S&D).

## Appendix A

**Table A1:** Coding scheme.

Category Label	Category Definition	Anchor Example	Coding Rules
1 pro regulation			
1 A pro regulation	Explicit expression of opinion pro regulation without providing specific reasons	“So it is great to see that commitment from the Commission, and leafing through the strategy, I must say the analysis is very strong, the language is right and the intentions are very, very promising.”	An explicit statement in favor of regulation without providing specific reasons or providing a reason which is not covered by one of the other categories under (1) below.
1 B pro regulation due to environmental concerns	Explicit expression of opinion pro regulation, reason(s) provided including ingestion, entanglement, habitat damage, climate impacts	“It seems to me that we’re moving from landfills to sea fills, and that’s really serious because of the impact on our ecosystems. Therefore, this strategy is vital, and I thank both Commissioners for being here this evening to present it.”	An explicit statement including environmental concerns and explicit approval of regulation (at least) somewhere in the whole statement. Only coded once per paragraph. If another reason is given in the same sentence, both this category and the other category are selected.
1 C pro regulation due to human health concerns	Explicit expression of opinion pro regulation, reason(s) provided including food safety, chemical exposure, poorly managed waste	“Plastic pollution is an enormous challenge, threatening our environment and health.” [also coded as 1 B]	An explicit statement including human health concerns and explicit approval of regulation (at least) somewhere in the whole statement. Only coded once per paragraph. If another reason is given in the same sentence, both this category and the other category are selected.
1 D pro regulation due to economic considerations	Explicit expression of opinion pro regulation, reason(s) provided including business opportunities and the prevention of (future) loss of revenue	“With this strategy, we aim to foster a new, sustainable and profitable plastics economy, with the design of products and other parts of the production chain taking into account re-use and recycling needs.”	An explicit statement including favorable economic impacts and explicit approval of regulation (at least) somewhere in the whole statement. Only coded once per paragraph. If another reason is given in the same sentence, both this category and the other category are selected.
1 E pro regulation due to non-use values	Explicit expression of opinion pro regulation, reason(s) provided including existence value, bequest value and altruistic value	“Wir haben auch das Problem, dass wir die Rohstoffe unserer Kinder verbrauchen…“ “We need an ambitious goal to save the next generation from drowning in a new ocean of waste.”	An explicit statement including non-use value(s) and explicit approval of regulation (at least) somewhere in the whole statement. Only coded once per paragraph. If another reason is given in the same sentence, both this category and the other category are selected.
2 pro regulation with reservations			
2 A pro regulation with reservations	Explicit expression of opinion pro regulation, but with certain reservations	“That is not to say that I do not think we need any—of course we do, we do need a framework—but I would like to make an appeal for that to be as light as possible in the sense of setting Member States’ targets and allowing them to use their own best efforts to meet those.” [condition other than below]	An explicit statement in favor of regulation with reservations that are not specifically stated or providing a reason which is not covered by one of the other categories under (2) below.
2 B pro regulation, but regulation should focus on economic incentives	Explicit expression of opinion pro regulation, but with strong focus on economic incentives instead of regulative measures		An explicit statement favoring economic incentives over regulative measures and explicit approval of regulation (at least) somewhere in the whole statement. Only coded once per paragraph. If another reason is given in the same sentence, both this category and the other category are selected.
2 C pro regulation, but additional measures necessary	Explicit expression of opinion pro regulation, but with an emphasis on the need of additional measures	“I’m very happy with the strategy, but I had expected a little bit more on the legislative part. I know that your intention is to come forward with those, please do so during your mandate because if it is left to the next Commission we don’t know if the same two vice-presidents will still be still there.”	An explicit statement emphasizing that additional measures should be taken and explicit approval of regulation (at least) somewhere in the whole statement. Only coded once per paragraph. If another reason is given in the same sentence, both this category and the other category are selected.
3 contra regulation			
3 A contra regulation	Explicit expression of opinion contra regulation without providing specific reasons		An explicit statement against regulation without providing specific reasons or providing a reason which is not covered by one of the other categories under (3) below.
3 B contra regulation due to lack of evidence to cause environmental harm	Explicit expression of opinion contra regulation, reasons including total lack of evidence for environmental harm and skepticism towards existing research findings		An explicit statement including lack of evidence with regards to environmental harm and explicit rejection of regulation (at least) somewhere in the whole statement. Only coded once per paragraph. If another reason is given in the same sentence, both this category and the other category are selected.
3 C contra regulation due to lack of evidence to cause harm to human beings	Explicit expression of opinion contra regulation, reasons including total lack of evidence for harm to human beings and skepticism towards existing research findings		An explicit statement including lack of evidence with regards to harm to human beings and explicit rejection of regulation (at least) somewhere in the whole statement. Only coded once per paragraph. If another reason is given in the same sentence, both this category and the other category are selected.
3 D contra regulation due to economic considerations	Explicit expression of opinion contra regulation, reasons including competitive disadvantages, increase in consumer prices		An explicit statement including unfavorable economic impacts and explicit rejection of regulation (at least) somewhere in the whole statement. Only coded once per paragraph. If another reason is given in the same sentence, both this category and the other category are selected.
3 E contra regulation as regulation does not address problem in the right way	Explicit expression of opinion contra regulation, reasons including wrong choice of instrument(s), only “symbolic policies”	“Das konzeptionelle Korsett für diese Strategie wird sich jedoch kaum als tragfähig erweisen, denn die gewählte Freiwilligkeit, der Fokus auf Recycling und Wiederverwertung sowie die Betrachtung des Plastikproblems allein aus der Sicht des Binnenmarkts sowie von Angebot und Nachfrage werden keine Lösung liefern.”	An explicit statement highlighting that the problem should be addressed differently and explicit rejection of regulation (at least) somewhere in the whole statement. Only coded once per paragraph. If another reason is given in the same sentence, both this category and the other category are selected.

**Table A2:** Results of the qualitative content analysis: Number of coding units per sub-category and parliamentary group/committee/European Commission/Council.

	1 A	1 B	1 C	1 D	1 E	2 A	2 B	2 C	3 A	3 B	3 C	3 D	3 E	∑
ALDE	4	8	3	4	2	1	0	7	0	0	0	0	1	30
ECR	5	8	2	10	0	1	0	3	0	0	0	0	0	29
EFDD	5	6	2	1	0	0	0	2	1	2	0	5	2	26
ENF	4	3	4	1	0	1	0	1	4	2	0	4	4	28
EPP	22	21	7	10	8	16	0	23	0	0	0	0	4	111
The Greens/EFA	9	15	10	6	1	0	0	9	0	0	0	0	0	50
GUE/NGL	6	9	1	0	0	10	0	7	0	0	0	0	1	34
S&D	19	25	13	14	2	2	1	13	0	0	0	1	3	93
AGRI committee	2	0	0	0	0	0	0	1	0	0	0	0	0	3
ENVI committee	0	0	0	0	0	0	0	1	0	0	0	0	0	1
PECH committee	3	3	1	1	0	0	0	1	0	0	0	0	0	9
European Commission	12	8	3	16	0	0	0	1	0	0	0	0	0	40
Council	1	0	0	0	0	1	0	0	0	0	0	0	0	2
**∑**	92	108	47	65	13	32	1	70	5	4	0	10	16	463
